# Transcranial direct current stimulation as treatment for major depression in a home treatment setting (*HomeDC* trial): study protocol and methodology of a double-blind, placebo-controlled pilot study

**DOI:** 10.1186/s40814-023-01423-x

**Published:** 2023-12-15

**Authors:** Ulrike Kumpf, Harry Ezim, Matthias Stadler, Gerrit Burkhardt, Ulrich Palm, Esther Dechantsreiter, Frank Padberg

**Affiliations:** 1 Department of Psychiatry and Psychotherapy, Ludwig Maximilians University Munich, Nussbaumstr. 7, 80336 Munich, Germany; 2 Faculty of Psychology and Educational Sciences, Ludwig Maximilian University Munich, Munich, Germany; 3 Medicalpark Chiemseeblick, Bernau-Felden, Germany

**Keywords:** Non-invasive brain stimulation, tDCS, Major depressive disorder, Home-based treatment, Home treatment, Remote control

## Abstract

**Introduction:**

Transcranial direct current stimulation (tDCS) of prefrontal cortex regions has been reported to exert therapeutic effects in patients with major depressive disorder (MDD). Due to its beneficial safety profile, its easy mode of application, and its cost-effectiveness, tDCS has recently been proposed for treatment at home. This would offer new chances for regionally widespread and long-term application. However, tDCS at home must meet the new methodological challenges of handling and adherence. At the same time, data from randomized controlled trials (RCT) investigating this mode of application are still lacking. In this pilot RCT, we therefore investigate the feasibility, safety, and effectiveness of a new antidepressant tDCS application set-up.

**Methods and analysis:**

The *HomeDC* trial will be conducted as a double-blind, placebo-controlled, parallel-group design trial. Thirty-two study participants with MDD will be randomly assigned to active or sham tDCS groups. Participants will self-administer prefrontal tDCS for 6 weeks. Active tDCS will be conducted with anode over F3, cathode over F4, for 5 sessions/week, with a duration of 30 min/day, and 2 mA stimulation intensity. Sham tDCS, conversely, follows an identical protocol in regard to electrode montage and timing, but with no electric stimulation between the ramp-in and ramp-out periods. Both conditions will be administered either as a monotherapy or an adjunctive treatment to a stable dose of antidepressant medication. Adjunctive magnetic resonance imaging (MRI) and electric field (E-field) modelling will be conducted at baseline. Primary outcome is feasibility based on successfully completed stimulations and drop-out rates. The intervention is considered feasible when 20 out of 30 sessions have been fully conducted by at least 75% of the participants. Effectiveness and safety will be assessed as secondary outcomes.

**Discussion:**

In the *HomeDC* trial, the technical requirements for a placebo-controlled tDCS study in a home-based treatment setting have been established. The trial addresses the crucial points of the home-based tDCS treatment approach: uniform electrode positioning, frequent monitoring of stimulation parameters, adherence, and ensuring an appropriate home treatment environment. This study will further identify constraints and drawbacks of this novel mode of treatment.

**Trial registration:**

www.ClinicalTrials.gov. Trial registration number: NCT05172505. Registration date: 12/13/2021.

## Background

Major depressive disorder (MDD) is a disorder with a high prevalence. It is one of the leading causes of work disability worldwide [1]. For the treatment of MDD, psychotherapy and/or pharmacotherapy are recommended, depending on the severity of the disease. While pharmacotherapy is available almost everywhere, it is not suitable for all patients and is sometimes associated with considerable side effects or even intolerance [2]. Psychotherapy is not always readily available. Furthermore, approximately 33% of patients suffering from MDD have a treatment-resistant course even after guideline-appropriate stepwise therapy and do not respond adequately to pharmacological interventions [3].

Possible non-drug therapies for MDD include non-invasive transcranial brain stimulation (NIBS) techniques. NIBS techniques are well established in experimental neurosciences and have been increasingly used in recent years in the treatment of psychiatric diseases, especially depressive disorders [4, 5].

Studies on the antidepressant efficacy of transcranial direct current stimulation (tDCS) show promising, but not fully consistent, results. Two large, randomized, double-blind, placebo-controlled trials [6, 7] demonstrated significant antidepressant effects within several weeks of treatment with tDCS compared with sham stimulation. Meta-analyses on the topic also confirm the efficacy of tDCS over placebo in the treatment of MDD [8, 9].

The major advantage of tDCS over the other main NIBS technique, transcranial magnetic stimulation (TMS), is its applicability in a wide range of settings. From its use in magnetic resonance imaging (MRI) scanners to its therapeutic application as a treatment at home, the latter having been poorly investigated in studies thus far [10]. TDCS also has a very favourable side effect profile with a low incidence of complications [11]. The possibility of tDCS home-based treatment has already been discussed for some years now, and solutions for the technical requirements of its implementation have been developed and presented [12, 13]. At home tDCS treatment is convenient, given limited psychotherapy availability for MDD, as well as the current pandemic, COVID-19, also limiting frequent clinic contact.

So far, the few studies and case reports published on tDCS treatment at home have mainly investigated its neurological applications [14–16], for example, the treatment of cognitive impairment and fatigue in Parkinson’s disease, cerebellar ataxia, chronic pain, fibromyalgia, tinnitus, and in apallic patients [13–19]. In sum, results of the described case reports show good feasibility. Severe side effects were not reported, with larger case series showing a satisfactory degree of safety [20]. Reviews on the topic confirm these findings and highlight new monitoring opportunities [10, 21]. No serious therapeutic side effects were found in the 19 studies reviewed by Sandran et al. [21].

In the psychiatric field, two patients with paranoid schizophrenia had successful partial remission of previously treatment-resistant multimodal hallucinations using at home tDCS [22, 23]. These aforementioned studies represent the majority of research on at home tDCS, which is mostly centered around neurological disorders and certain psychiatric conditions. In the following, we will highlight the few published studies examining the treatment of depression with at home tDCS. One of these few studies is a placebo-controlled trial investigating the home-based application of prefrontal tDCS in 26 participants. These participants presented with temporal lobe epilepsy and depressive symptoms. There was a total of 20 sessions, 20 min each, at an intensity of 2 mA. Results showed good feasibility, but no significant difference between the groups in regard to the reduction of depressive symptoms [24]. In another home-application MDD case series, 12 trial participants with treatment-resistant, chronic depression were treated with tDCS. This served partly as maintenance therapy after electroconvulsive therapy (ECT) or repetitive TMS. After at least 6 weeks of treatment, significant improvements in depressive symptoms were observed. The effects lasted in part for several months [25]. In an open label, home-based tDCS trial by Alonzo and colleagues [26], with the same protocol as *HomeDC*, 34 trial participants with unipolar and bipolar depression conducted a minimum of 24 tDCS sessions. Overall, this pilot trial addressed the issues of electrode positioning, supervision, training, and control of adherence. There was improvement in depressive symptomatology (response rate of 38%), the drop-out rate was very low at 6%, and side effect outcomes were similar to tDCS studies in a clinic setting [26]. However, a control condition was lacking in this pilot study. Another case series combined home-based tDCS (bifrontal montage, 21 sessions over 6 weeks) with app-based psychological intervention in five participants with MDD [27]. Safety and feasibility were good with a response rate of 80%, even in combination with the app-based intervention. Technical parameters were not monitored. In the largest home-based tDCS trial for MDD to date, a placebo-controlled, single-blind design was used to stimulate the prefrontal cortex in 58 trial participants with MDD (2 mA, 30 min, 6 weeks) [28]. Results showed feasibility and a significant reduction in the Beck Depression Inventory (BDI) in the active tDCS group. Some methodological limitations, e.g., the single-blind design and simultaneous administration of varying escitalopram dosages have to be considered. In another small pilot study, ultimately, only three participants underwent the study protocol investigating the safety and feasibility of a home-based, “study companion-administered” tDCS intervention [29]. Another very recent study confirmed the feasibility and safety of home treatment with tDCS in an open label design without placebo control. Here, the same parameters as the *HomeDC* trial were used for 6 weeks in 26 participants. Antidepressant effects were promising and long lasting with 91.3% clinical response and 73.9% remission rates even at the 6-month follow up [30].

Looking forward, we await the findings of further studies. The study protocols of these larger randomized controlled trials (RCT) have already been published, partly combining tDCS with cognitive training and behavioral therapy. The results of these specific trials are expected in future and will be a welcome addition to the results of the pilot studies, case reports, and smaller monocentric single-blind trials highlighted above [31, 32].

The following guidelines for the home-based use of tDCS helped navigate issues encountered during the design of the *HomeDC* study [33]:

1. Training and supervision. A checklist [26] and instructional videos are helpful for making training easier, standardizing procedures, and play an important role in tele-health solutions [34]. The common usage of assisted home treatment via a caregiver or family member appears effective in treating physically impairing or neurological diseases [27]. This approach however is not conducive to the treatment of MDD. As the goal is to save resources and increase patient autonomy at home.
2. Electrode positioning. Different technical solutions were tested and have been selected to combine the highest possible degree of accuracy and individuality with ease of user [22, 23, 35–38].
3. Monitoring adherence to the tDCS protocol. Here, we find various options ranging from personal consultations and pre-programmed block times to monitoring stimulation times and duration.
4. Safety monitoring and assessment of targeted outcomes. Outcomes in the psychiatric field are most accurately measurable through personal ratings. Monitoring technical stimulation parameters could contribute to safety monitoring and stimulation quality. A more personnel-intensive option would be assisted stimulation.

Grounded in these critical points, particular strengths of the *HomeDC* trial design are cloud-based supervision of technical data and timing, new caps offering standardized electrode positioning, and individualized dosage through approximated modeling of electric field (E-field) intensity based on individual MRIs.

With cloud-based monitoring and supervision of the technical data, the study provides a contribution to technical achievements, which will allow us to draw objective conclusions about the quality of the stimulations performed. Variability in impedance, current, and voltage will be correlated with clinical outcome to find further possible factors influencing the effectiveness of tDCS. It will also be possible to compare the technical data of the *HomeDC* trial with that of the *DepressionDC* trial, a large scale RCT covering in-clinic tDCS in MDD [39]. Thus, contrasting stimulation quality between home-based, self-administered tDCS and on-site application by an operator (*DepressionDC*). The time tag of the stimulations will be used to check the adherence.

For correct and easy electrode montage in the *HomeDC* trial, caps with integrated electrodes have been developed. Unlike most contemporaries, these take into account not only the head circumference, but also the distance between the external eye angle and the Cz electrode point; as the exact positioning of the electrodes is crucial for correct tDCS stimulation of the target [40].

Using these standards in a placebo-controlled, double-blind, randomized design, the *HomeDC* trial aims to investigate the feasibility, safety, and effectiveness of prefrontal tDCS as an at home treatment for MDD. In addition, the *HomeDC* trial adopts and tests a cloud-based approach for supervising timing and technical data, recently innovated by the *DepressionDC* trial [39].

## Methods

### Design

**Fig. 1:**
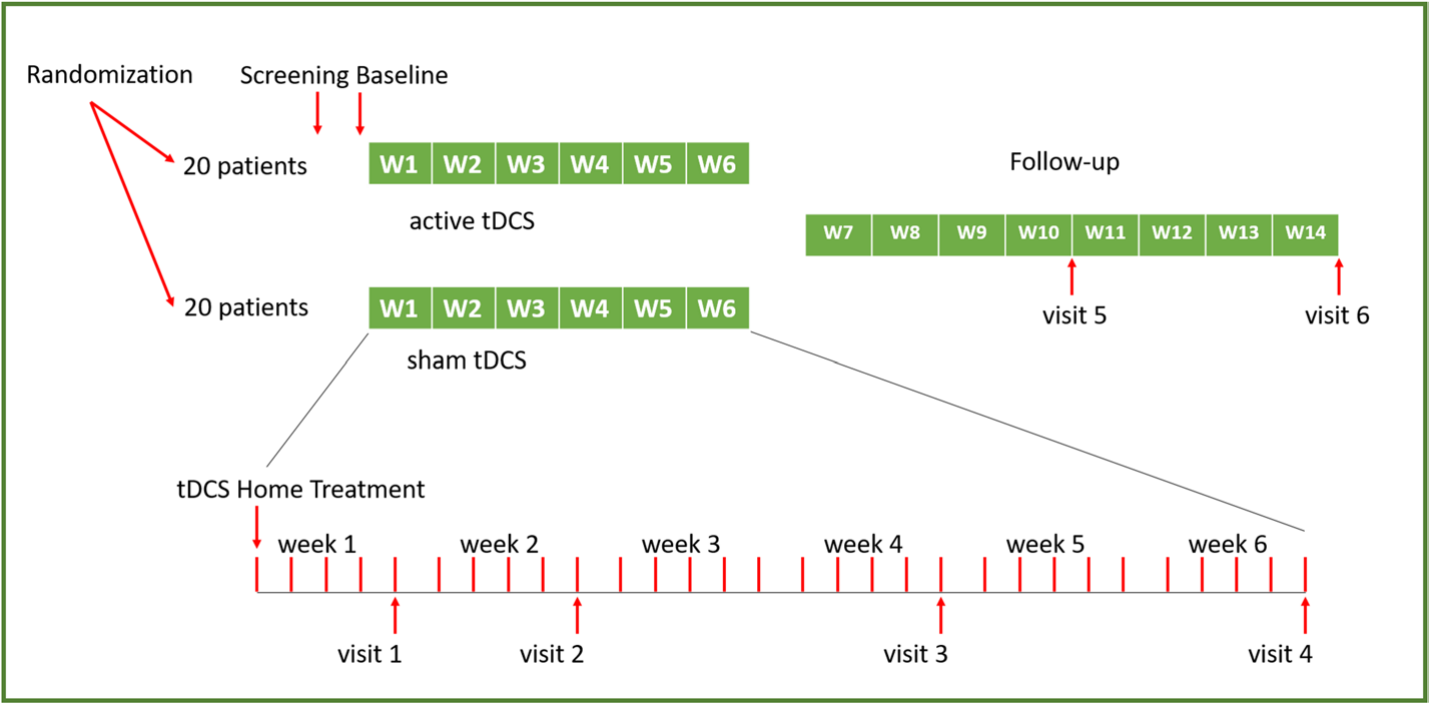
Study design

The study will be conducted in a double-blind, placebo-controlled, parallel-group design with 20 study participants per group. This trial will take place at the Department of Psychiatry and Psychotherapy of the Ludwig-Maximilians-Universität Munich (LMU). Participants with a primary MDD diagnosis according to the Diagnostic and Statistical Manual of Mental Disorders, 5th Edition (DSM-5), will perform a 6-week self-administered treatment with prefrontal active tDCS or sham tDCS at home; either as a monotherapy or as an adjunctive treatment to stable antidepressant medication. Participants will be divided (1:1) into two groups by fixed-blocked randomization, each group receiving either active tDCS or sham tDCS for a total of 6 weeks (5x/week, maximum 30 sessions). At baseline, optional cranial MRI (cMRI), consisting of structural MRI (sMRI) and functional MRI (fMRI), will be performed. E-field modeling, based on the cMRI scans, may also take place. During the treatment phase, a study visit will take place every 2 weeks. An additional study visit will take place after the first week addressing any difficulties with self-application at home. After 6 weeks, the primary endpoint will be reached concluding the treatment phase. The final rating will be made in the ensuing follow-up phase, consisting of study visit 5 and 6 (V5) (V6). V5 and V6 will take place 4 weeks and 8 weeks, respectively, after the last stimulation session (Fig. 1). According to the *DepressionDC* trial [41], a total of 4 sessions may be missed without denoting a drop-out. Missed sessions can be compensated with additional sessions in week 7. All devices used in this study have CE certifications.

### Study objectives

The present project aims to investigate the feasibility, effectiveness, and safety of bifrontal tDCS as an at home treatment approach to MDD in a placebo-controlled, parallel-group design. The study objectives regarding feasibility consist of demonstrating that a 6-week home-based stimulation is feasible for patients with MDD.

The primary outcome is feasibility based on successfully completed stimulations and drop-out rates. *HomeDC* considers the intervention to be feasible when 20 out of 30 sessions have been fully conducted by at least 75% of the participants. This definition of testing feasibility is the same as a recent trial protocol, the *DiSCoVeR* trial [31]. *DiSCoVeR* set its target to a minimum of 50%, due to its combination of tDCS and gamification applications. Using this framework, *HomeDC* chose a target between 50% and the ceiling value of over 90% reported in aforementioned trials [26, 30]. Secondary outcomes are safety, based on side effects and complications encountered, as well as stimulation discontinuation rates due to high impedances. Safety is measured based on the number of adverse events (AE) or serious adverse events (SAE) as well as adverse device effects (ADE) and serious adverse device effects (SADE). A comfort rating questionnaire (CRQ) will be completed by the study participants after each session as a safety measurement tool [42]. On a scale from 1 to 10, participants note perceived subjective intensity of brain stimulation relevant side effects. This self-report questionnaire allows the research team to detect and follow up on any AEs, as well as monitor tolerability. Effectiveness is measured by a reduction in the Montgomery and Asberg Depression Rating Scale *(*MADRS) after 6 weeks of at-home tDCS treatment. We will use MADRS scores to calculate response and remission rates in both groups. Response is defined as a reduction ≥ 50% in MADRS score and remission as MADRS score < 11 [43].

**Fig. 2:**
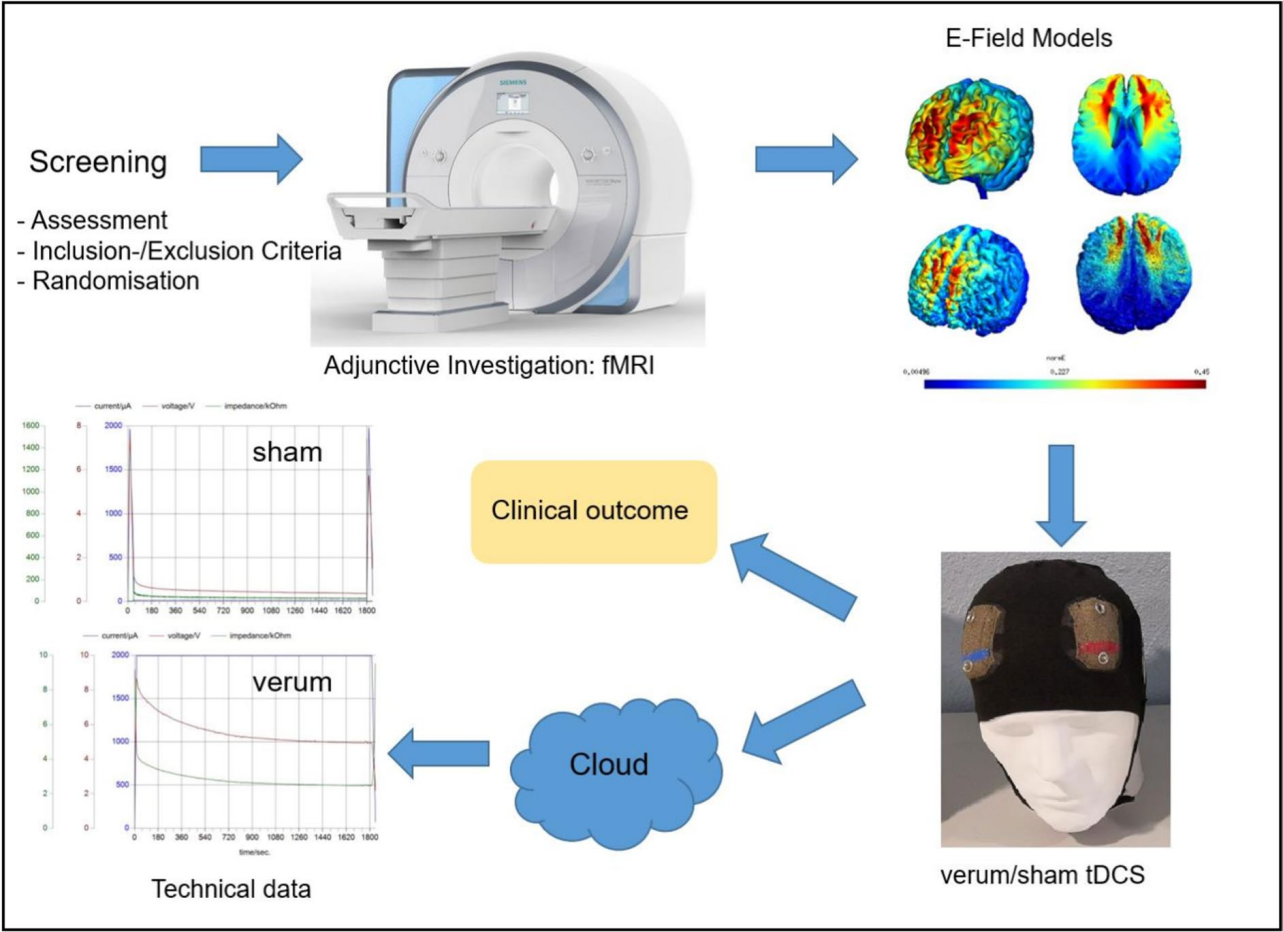
Technical workflow

Further outcomes are the changes in the Beck Depression Inventory (BDI), the General Assessment of Functioning (GAF), and the Clinical Global Impression-Improvement/-Severity (CGI-I/-S) scores. In both active and sham tDCS groups, baseline measurements will be compared to measurements documented at the primary endpoint after 6 weeks and during the follow-up visits. Stored data on timing, impedance, voltage, and current flow will be transmitted to measure stimulation quality (Fig. 2).

### Participants

Potential study participants, presenting with MDD, are recruited from the brain stimulation outpatient clinic of the LMU and through local advertising (each approved by the ethics committee). Patients of the psychiatric day-care hospital of the LMU may be approached by recruitment members of the research team. State of research, background, and possible benefits and risks of tDCS, as well as the design of the study are thoroughly explained. Potential participants are specifically informed that they may receive sham tDCS and that an improvement of their MDD symptoms cannot be guaranteed. Potential participants may take as much time as they need to decide whether they are willing and able to take part in the study. To this effect, they will be given the contact information of a member of the research team in case they have any more questions and/or decide to participate. After reaching out, all potential participants are given an appointment for eligibility screening. Study participants receive a cost reimbursement of 55 euros for travel expenses. All study participants have complete insurance for study procedures and travel to the study center through ECCLESIA mildenberger HOSPITAL GmbH.

#### Eligibility criteria

- Males and females between 18 and 70 years of age
- Primary diagnosis of unipolar Major Depressive Episode (single or recurrent) according to DSM-5 criteria as assessed by the Mini International Neuropsychiatric Interview (M.I.N.I) [44]
- Current episode present for at least 4 weeks, but lasting no longer than 5 years, at the time of study inclusion (in between episodes there must be a period of ≥ 2 months, in which the participant did not meet full criteria for the DSM-5 definition of major depressive episode).
- Total score ≥ 13 in the Hamilton Depression Rating Scale (HDRS-17) [45] during screening
- No medication or stable medication for at least 2 weeks at time of screening. Allowed medications are (also in combination): selective serotonin reuptake inhibitors (SSRIs), selective serotonin–norepinephrine reuptake inhibitors (SSNRIs), serotonin–norepinephrine–dopamine reuptake inhibitors (SNDRIs), Mirtazapine, Vortioxetine, Agomelatine, Trazodone, Tianeptine, Opipramol, Moclobemide, and tricyclic antidepressants.

In addition, a stable dosage of an augmentative combination (antidepressant base drug) for at least 2 weeks is also allowed as follows: Quetiapine, Aripiprazole, Risperidone, and Olanzapine.

Other antipsychotics may also be combined if the dose is stable for at least 2 weeks prior to the start of treatment. They must not reach the threshold of the minimally effective dose according to the antidepressant treatment history form (ATHF). Combination with lithium is allowed if lithium dosage is stable for at least 3 months prior to study inclusion.

Rescue medication with Zopiclone (on demand up to 7.5 mg/day orally), Zolpidem (up to 10 mg/day), and Benzodiazepine up to a dose equivalent to 2 mg of Lorazepam is allowed.

All medication taken by a study participant during treatment and/or taken up to at least 4 weeks before treatment start must be documented. During the course of the trial (treatment and follow-up phase), the medication dose should not be changed.

- Concomitant psychotherapy is permitted. Type, modality (e.g., group vs. individual therapy), duration, and frequency of therapy must be accurately documented.
- Participant is capable and willing to provide informed consent.

*Exclusion criteria*

- Acute risk for suicide, defined as a score of 4–10 on item 10 of the MADRS [46], or attempted suicide in the current episode
- Any relevant psychiatric axis-I- and/or axis-II-disorders other than MDD assessed by M.I.N.I. to be the primary diagnosis. Depression as a secondary disease to another disease: e.g., psychotic disorder (current), bipolar disorder (lifetime), eating disorder, obsessive–compulsive disorder, post-traumatic stress disorder, generalized anxiety disorder, panic disorder, social phobia, personality disorder, and substance dependence in the past 6 months (nicotine and caffeine excluded).
- Any relevant neurological disorder including increased intracranial pressure, space-occupying brain lesions, history of cerebrovascular accidents, transient ischemic attacks within the last 2 years, cerebral aneurysm, dementia, Parkinson’s disease, Huntington’s chorea, multiple sclerosis, and epilepsy (including history of seizures).
- ECT in the current episode
- History of tDCS, except for single tDCS sessions during experimental studies
- Intracranial implants or any other intracranial metal objects (excluding the mouth) that cannot be safely removed
- Confirmed pregnancy (according to pregnancy test at baseline visit)
- Treatment with deep brain stimulation or vagus nerve stimulation
- Any relevant unstable medical condition
- Site personnel and investigators, directly affiliated with this study

### Power analysis and sample size calculation

Considerations of adequate sample size for feasibility outcomes draw on previous study results, with sample sizes varying from case series [27, 45] to larger randomized controlled trials with up to 64 participants [28, 47]. The dropout rate or rate of missed stimulations also varies depending on the study protocol. A similar study protocol with only 4 weeks of stimulation plus taper phase (32 stimulations in total) was used by Alonzo and colleagues [26]. This study, albeit without sham control, involved 34 participants and resulted in a drop-out rate of 6% with 93% of sessions completed. Similar numbers were found in the trial of Woodham and colleagues. Of 26 participants included, 92.3% completed the 6-week treatment phase [30]. Thus, with respect to feasibility, a comparable sample was intended.

Regarding the antidepressant effects, a power calculation was performed based on the amputated intention-to-treat (ITT) sample of the SELECT trial [7]. The data set from this trial was available to our group for sample size calculation. The change in MADRS score between the placebo-medication/sham tDCS condition and the sertraline (50 mg)/active tDCS condition was used. A change in the MADRS score was hypothesized because the effects of tDCS are thought to be synergistic with existing serotonergic medication [48]. In this pilot study, participants should have been on stable medication for at least 2 weeks or be medication-free. Thus, at baseline, they had not yet responded adequately to existing medication as seen in a minimum HDRS-17 score of 13. Only active tDCS is expected to have a synergistic effect [49]. Since the 50 mg sertraline was a very low dose, the synergistic effect in the SELECT study [7] is questionable, but it can be assumed that study participants with stable serotonergic medication get such an effect, so that with the assumption of the effects of the two groups the achieved effect is not overestimated.

The difference of change in MADRS scores (pre-post treatment phase) between the two groups was *M* = 12.58 points. The standard deviation of the change was SD = 11.01 points. After week 6, this leads to a Cohen’s *d* of 1.14. With power = 0.90 and a two-sided test, the G*Power analysis results in a required participant number of 18 per group.

Assuming a significance level of *α* = 0.05 and power = 0.90, a total sample size of 36 study participants would detect a true group difference of 12.58 MADRS points. Further taking a drop-out rate of approximately 10% into account leads to a total recruitment number of 40 study participants.

### Interventions and procedures

TDCS will be performed according to the protocol of the *DepressionDC* trial [41] with the total number of stimulations increased to 30 sessions in order to achieve longer lasting effects. With more than 3200 completed stimulations, the protocol has proven itself credible in application and implementation. Participants will self-apply 5 tDCS sessions per week (Monday to Friday) for 6 weeks at home. Electrode montage will be bifrontal with the anode over F3 and the cathode over F4 (international 10–20 electroencephalogram system). Electric stimulation will be held at 2 mA in the active tDCS group. Each stimulation will last 30 min, with electric current ramp-in (15 s) and ramp-out (30 s) phases beginning and ending each session. The sham group will have ramp-in and ramp-out phases analogous to the active tDCS group. In contrast to the active group, the electric current will be turned off in between these phases during the 30-min sham stimulation period. To make positioning the electrodes at home easier and to avoid errors, a special stimulation cap will be used. This innovative cap, made by neuroConn (neuroCare Group, Illmenau, Germany), utilizes built-in electrodes over the corresponding target areas [50].

**Fig. 3:**
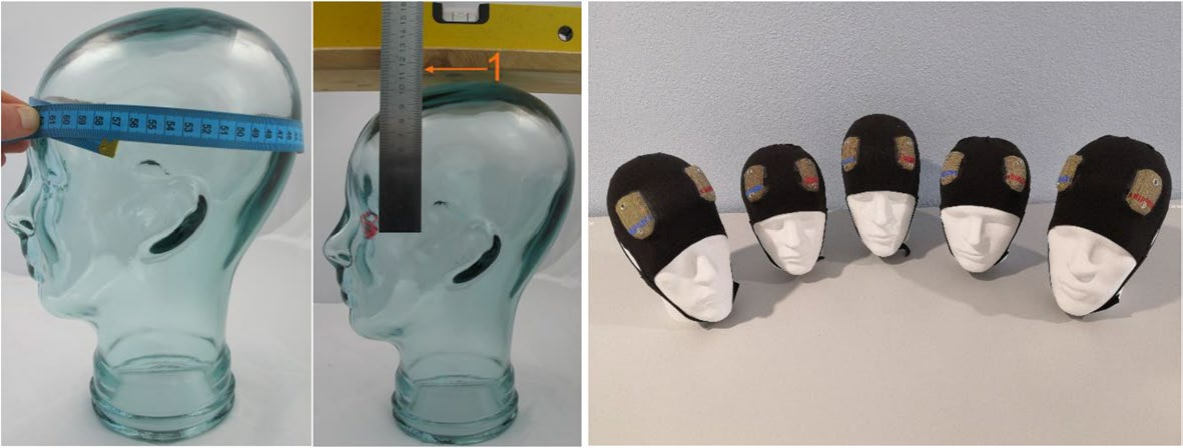
Measurement of head circumference and distance between eye angle and vertex (1). Five cap sizes for different head shapes

Five different manufactured cap sizes ensure correct electrode positioning for all head shapes (Fig. 3). At the screening visit, the head circumference as well as the distance between vertex and external eye angle will be measured (head position in the “Frankfurter Horizontale”; participant looks straight ahead into the distance). The appropriate cap is selected to match the participant’s individual anatomy. Using a syringe, a maximum of 20 ml of NaCl will then be injected into the opening provided for each electrode.

*HomeDC* will use the same CE-certified stimulators from neuroConn as the *DepressionDC* trial. The stimulators and software were designed specifically for *DepressionDC* to ensure double-blinding (investigator and participant). Impedance, voltage, and current will be measured per second. This technical data will be recorded, stored, and transferred analogous to the *DepressionDC* trial [37]. For safety, an impedance control lock prevents stimulation at electrode impedances > 55 kOhm in accordance with the *DepressionDC* study. In addition, a pre-programmed safety mechanism only allows stimulation once a day. Stimulation is automatically locked for 16 h between sessions. After each treatment, participants also complete a CRQ to record potential side effects or AEs. These CRQS are monitored by the research team, in case they warrant further investigation and/or suspension of the treatment phase.

The CE-certified stimulators are charged with 10 stimulations at baseline, V1, V2, and V3. These stimulators are pre-programmed with either verum or sham codes that decide whether the stimulations are active or sham. The codes are randomized pre-trial into a list using an online tool by neuroCare (https://ctrandomization.cancer.gov/tool/). Each participant is assigned a code from the randomized list in order of trial enrolment date. This assignment of either a verum or sham code from the randomized list is performed by an independent researcher not otherwise involved in the study. The participants are not aware of their codes. Neither the operator nor the rater knows whether the individual stimulation ID codes are verum or placebo. As no conclusions can be made on the stimulation condition, double-blinding is ensured [41].

### Assessments

The time and events schedule (Table 1) summarizes the frequency and timing of treatment and study assessments. At screening, each participant will undergo a diagnostic interview using M.I.N.I. A clinician, who is familiar with the DSM-5 classification and diagnostic criteria will perform the interview. The M.I.N.I ensures that study participants meet the diagnostic criteria for an episode of MDD according to DSM-5. Efficacy will be assessed using the MADRS (primary outcome) at 8 timepoints. Further assessments such as the BDI, CGI-I/-S, and GAF also take place every visit. Additionally, at the baseline visit, the Edinburgh Handedness Inventory (EHI) and ATHF will be assessed. Afterwards, tDCS training will take place. During the treatment period, CRQs are turned in to the research team each visit for safety and tolerability evaluation.

**Table 1:** Times and events schedule

**Phase**	**Pre-treatment period**		**Treatment period**																														**Follow-up period**	
**Week**	** − 1**		**1**					**2**					**3**					**4**					**5**					**6**					**10**	**14**
**Study visit**	**S**	**B**					**1**					**2**										**3**										**4**	**5**	**6**
**Day**	-14/-5	-4/1	1	2	3	4	5					12					19					26					33					40	68	96
**Screening, treatment, and administrative procedures**																																		
Informed consent^a^	**√**																																	
Medical and psychiatric (family) history, demographic data	**√**																																	
M.I.N.I	**√**																																	
HDRS-17	**√**																																	
Pregnancy test		**√**																																
Concomitant treatment^b^		**√**																														**√**		
EHI-8, ATHF		**√**																																
tDCS training		**√**																																
Blinding check																																**√**		
**Efficacy measures**																																		
MADRS	**√**	**√**					**√**					**√**										**√**										**√**	**√**	**√**
BDI-II, CGI, GAF,		**√**					**√**					**√**										**√**										**√**	**√**	**√**
**Safety measures**																																		
CRQ			**√**	**√**	**√**	**√**	**√**	**√**	**√**	**√**	**√**	**√**	**√**	**√**	**√**	**√**	**√**	**√**	**√**	**√**	**√**	**√**	**√**	**√**	**√**	**√**	**√**	**√**	**√**	**√**	**√**	**√**		
**Adjunctive investigations**^e^																																		
fMRI, sMRI, E-field Modelling		**√**																																

^a^Informed consent has to be obtained prior to any study-related procedure

^b^Medication, but also the kind of psychotherapeutic intervention (e.g., group therapy or individual therapy), duration, and frequency have to be recorded

Assessments during visit 4 (week 6, post-intervention) are crucial in case of premature discontinuation of the study. Participants will drop out of the study if there is attempted suicide or presentation of suicidal ideation (based on a score of 4 or higher in MADRS item 10).

### Magnetic resonance imaging (MRI)

A total of 20 participants can be offered optional cMRI, comprising of sMRI and fMRI scans, as two simultaneous additional baseline examinations prior to stimulation start. The sMRI scan would be performed for anatomical measurements and E-field modeling, whereas fMRI resting state scans, conducted at the same time, would supplementarily measure brain baseline activity through changes in the blood-oxgen-level-dependent signal (BOLD). The entire MRI protocol is expected to be approximately 30 min in length. Measurements will be performed on a Siemens Prisma 3 T MR scanner (3-T MAGNETOM Prisma, Siemens Healthineers, Erlangen, Germany) at the LMU Department of Psychiatry and Psychotherapy.

### Electric field modeling

Based on structural MRI measurements, models of tDCS-induced E-fields will be computed using SimNIBS 3.2.2., an open-source program (www.simnibs.de) [51, 52]. The intensity and shape of the E-field over the target region, the prefrontal cortex, are calculated from the anatomical scans of each respective participant. The characteristics of the E-field depend on, for example, the amount of cerebrospinal fluid and the distance between the electrode and the cortex. The different distribution of the E-fields could also explain interindividual differences in participants’ response patterns to tDCS.

### Safety and adverse events monitoring

The risk of (severe) adverse events or even health damage associated with prefrontal tDCS is expected to be minimal. Common adverse effects are generally mild [7]: skin irritation (22%), local sensations on the skin (40%), mild headaches (14%), and drowsiness (29%). After each treatment, participants complete a CRQ to record any adverse effects. These CRQS are monitored every study visit by the research team. A member of the study team will be available for any concerns until 8 pm via a study cellphone. For problems outside office hours, participants will be instructed to come to the psychiatric emergency department of the university hospital. Participants will be withdrawn from the study if there is any risk for suicidality (based on a score of 4 or more in MADRS item 10). Further reasons for withdrawal are pregnancy and SAE occurrence. If the investigators conclude that it is best for a participant to stop treatment for safety reasons, e.g., after an AE, the participant will be withdrawn from the trial. In these cases, unblinding is done by a scientist, who is not involved in the trial.

### Data management plan and statistics

The data will be collected and processed with appropriate precautions to ensure confidentiality and abide by data protection laws and regulations. Participants will be informed that all data will be appropriately pseudonymized and that this data may be used for analysis and publication purposes. Data analysis will be conducted using R software and IBM SPSS Statistics. Safety will be evaluated exploratively based on drop-out rates, technical parameters, and AEs. To evaluate the intervention’s feasibility, we will determine the mean number of sessions that were missed by participants. A 95% confidence interval will be computed around this mean to account for potential variability in this measure. Our predefined feasibility criterion for the intervention is met if, within the study, at least 75% of the participants fully complete 20 out of the 30 intervention sessions. To analyze the efficacy of the tDCS intervention, analysis of covariance (ANCOVA) will be used. The change in outcome (MADRS, BDI, CGI, and GAF) will form the dependent variable, treatment allocation (active vs. sham), and the fixed effect, and the baseline level of outcome will be entered as a covariate to control for baseline differences. This will reduce residual variability, increase the precision of the estimate, and address regression to the mean. The treatment effect will be reported with 95% confidence intervals.

## Discussion

The *HomeDC* trial is a randomized, placebo-controlled, double-blind clinical trial investigating the feasibility, safety, and efficacy of prefrontal tDCS for the treatment of MDD in a home treatment setting.

During the development of the *HomeDC* protocol, recent guidelines for the home use of tDCS [33] highlighted certain challenges that required careful consideration, especially in contrast to the use of tDCS in clinical settings. These challenges are as follows: electrode positioning, training study participants, frequent monitoring of stimulations and quality of electrode positioning, standardization and monitoring of the dose administered, ensuring an appropriate home treatment environment, and regular evaluation of target symptoms and any side effects. In addition, the implementation of a double-blind setup and an appropriate sham condition is crucial for the methodologically sound conduct of a RCT.

The pilot studies and case series on tDCS at home for MDD, previously mentioned in the “Background” section, have proposed some solutions to these issues. The different approaches and designs have their advantages and disadvantages. *Supervision of the home-based tDCS sessions* was done: via in-person monitoring (video call or direct) [28], via video link or Google Meets for the first sessions and then only on demand [24, 25], via on-demand remote assistance, and via joint sessions with a “study companion” [27]. The advantage of in-person monitoring is to ensure that tDCS is applied correctly and safely. The disadvantage is that a staff member must be present during each stimulation, requiring human resources. For tDCS training, a checklist of procedures was used to confirm participants’ ability to perform tDCS sessions independently [26]. Moreover, standardized training programs [29] and online manuals [28] were established in line with current guidelines and recommendations [33].

For the *HomeDC* trial, an effective and time-saving concept for both participant training and supervision was created. Standardized tDCS training will be performed in the clinic. Supervision will be enabled via telephone, with one team member carrying a study cellphone after hours until 8 p.m. each day in case technical problems occur and/or participants need advice. This will give trial participants greater agency over when their sessions occur, including outside office hours.

Regarding *self-administered electrode positioning*, different solutions have already been proposed and tested. Cappon and colleagues used a special cap. The general electrode placement was measured through an initial E-field calculation before the caps were manufactured. In addition, “optimized four-electrode montage” was established and tested in order to target the left dorsolateral prefrontal cortex [29]. Alonzo and colleagues used sponge electrodes, the positions of which were individually adjustable, although they required rather complex measurements [26]. Borrione and colleagues used a one-size-fits-all headset with circular electrodes covered by fixed sponge pads. Thus, the positioning of the electrodes was made easier and more practical [27]. The crux of self-administered electrode positioning is achieving maximum accuracy and individuality, while emphasizing user-friendliness and simplicity in order to avoid errors. The *HomeDC* trial proposes utilizing five different cap sizes as a solution to this issue. The caps are easy to use and respect individual anatomy [50].

*Monitoring of adherence, technical tDCS parameters, and side effect evaluation* can be addressed using a tablet-based system. This system would allow for remote side effects and compliance monitoring. Participants are able to report side effects via tablet. With this system, a stimulation slot can be scheduled, preventing unauthorized stimulation outside the times appointed [29]. Alonzo and colleagues overcame problems with adherence by requiring participants to enter a new stimulation code to begin their next stimulation. According to the study protocol, this code was sent only after the previous stimulation had been completed and the next stimulation was scheduled to begin [26]. An app-based control system that allowed monitoring stimulation duration, session intervals, and mean current intensity was also established by Oh and colleagues [28].

In the *HomeDC* trial, technical tDCS parameters will be monitored and controlled using the same equipment as in the *DepressionDC* trial. This will allow us to draw objective conclusions about the quality of the stimulations performed. High impedances would indicate a poorly executed montage, whereas impedance leaps during stimulation would indicate that electrodes, cables, or the stimulator were strained through external movement. Here, participants would be advised to adopt a quiet sitting position during stimulation. The time tags of the stimulations will be used to check adherence. Self-rated CRQs after each session serve to monitor side effects. This information is not automatically forwarded to the research team online via app. Rather, participants would need to call the study cellphone if they experience an AE. This lack of automation is a possible limitation of the trial.

Adherence is of great importance in the treatment of mental illness in general. In the case of home-based tDCS treatment, the importance of adherence cannot be overstated. Reliable, daily, accurate self-application is crucial for safety and for the treatment’s expected antidepressant effects. Overstimulation, poor stimulation quality or widely varying daily stimulation times will be monitored and, if possible, avoided.

The current results of at-home tDCS safety and feasibility trials are generally positive, even if inhomogeneous technical solutions were chosen to overcome the described pitfalls of a home-based treatment.

The studies published to date on home-based tDCS for depression offer good approaches to the issues mentioned. Double-blind RCTs are still lacking, as most of the published results in this field stem from case series or single-blind monocentric trials [28]. *HomeDC* implements a double-blind setup, already proven in the *DepressionDC* trial. Our trial submits far-reaching technical solutions for recording and analyzing tDCS parameters, facilitating future approaches to double-blind RCTs on home-based tDCS for MDD.

## Data Availability

The datasets used and/or analyzed during the current study are available from the corresponding author on reasonable request.

## Abbreviations

ADE: Adverse device effect
AE: Adverse event
ANCOVA: Analysis of covariance
ATHF: Antidepressant treatment history form
BDI: Beck Depression Inventory
BOLD: Blood-oxgen-level-dependent
CGI-I/-S: Clinical Global Impression-Improvement/-Severity
DSM-5: Diagnostic and Statistical Manual of Mental Disorders, 5th Edition
ECT: Electroconvulsive therapy
E-field: Electric field
EHI: Edinburgh Handedness Inventory
GAF: General Assessment of Functioning
HDRS-17: Hamilton Depression Rating Scale (17 Items)
ITT sample: Intention-to-treat sample
LMU: Ludwig-Maximilians-Universität Munich
MADRS: Montgomery and Asberg Depression Rating Scale
MDD: Major depressive disorder
M.I.N.I: Mini International Neuropsychiatric Interview
(c)MRI: (Cranial) Magnetic resonance imaging
(f)MRI: (Functional) Magnetic resonance imaging
S(MRI): (Structural) Magnetic resonance imaging
NIBS: Non-invasive transcranial brain stimulation
SSRI: Selective serotonin reuptake inhibitors
SSNRI: Selective serotonin–norepinephrine reuptake inhibitors
SNDRI: Serotonin–norepinephrine–dopamine reuptake inhibitors
SADE: Serious adverse device effect
SAE: Serious adverse event
(V1-6): Study visit 1–5
tDCS: Transcranial direct current stimulation
TMS: Transcranial magnetic stimulation
